# Chromosomal Radiation: A model to explain karyotypic diversity in cryptic species

**DOI:** 10.1590/1678-4685-GMB-2023-0116

**Published:** 2023-09-25

**Authors:** Karine Frehner Kavalco, Rubens Pasa

**Affiliations:** 1 Universidade Federal de Viçosa, Instituto de Ciências Biológicas e da Saúde, Laboratório de Genética Ecológica e Evolutiva (LaGEEvo), Campus Rio Paranaíba, Rio Paranaíba, MG, Brazil.; 2 Universidade Federal de Viçosa, Instituto de Ciências Biológicas e da Saúde, Laboratório de Bioinformática e Genômica, Campus Rio Paranaíba, Rio Paranaíba, MG, Brazil.

**Keywords:** Stasipatric speciation, chromosomal rearrangements, karyotypic polymorphisms, genomic plasticity, adaptive radiation

## Abstract

We present a concept that explains the pattern of occurrence of widely distributed organisms with large chromosomal diversity, large or small molecular divergence, and the insufficiency or absence of morphological identity. Our model is based on cytogenetic studies associated with molecular and biological data and can be applied to any lineage of sister species, chronospecies, or cryptic species. Through the evaluation of the karyotypic macrostructure, as the physical location of genes e satellites DNAs, in addition to phylogenetic reconstructions from mitochondrial and nuclear genes, per example, we have observed morphologically indistinguishable individuals presenting different locally fixed karyomorphs with phylogeographic discontinuity. The biological process behind this pattern is seen in many groups of cryptic species, in which variation lies mainly in the organization of their genomes but not necessarily in the ecosystems they inhabit or in their external morphology. It’s similar to the processes behind other events observed in the distribution of lineages. In this work, we explore the hypothesis of a process analogous to ecological-evolutionary radiation, which we called Chromosomal Radiation. Chromosomal Radiation can be adaptive or non-adaptive and applied to different groups of organisms.

## Stasipatric speciation and other models of chromosomal speciation

Since the 60’ the presence of chromosomal rearrangements in populations has been correlated with the origin of new species, mainly in organisms with restricted vagility. White et al. (1967) published the idea of a mechanism of chromosomal change acting on the emergence of species which was followed by many biologists. Over the years, besides the criticism, several authors claim that a range of organisms presenting different “chromosomal races” could evolve by the process proposed by White (1968), the Stasipatric Speciation (Shaw and Wilkinson, 1980; Watanabe and Kawanishi, 1983; Michailova, 1992; Frisman et al., 2009; Ruiz-García et al., 2011; López-López et al., 2021; Zamudio et al., 2023; among others). Stasipatric speciation is a model of non-allopatric speciation in which a chromosome rearrangement that reduces fitness when heterozygous is taken to be the post-mating isolating mechanism that confers species status on a population. Above all, the critics’ arguments have changed very little during this time. 

Probably, almost all cytogeneticists working with organisms with a high number of chromosomal karyomorphs faced a similar discussion that was pointed out primarily by Key (1968) in response to White et al. (1967). Key (1968) first argues that there was no evidence that speciation “*sensu stricto*” has occurred in *Vandiemenella*, a group of grasshoppers from Australia used as a model by White *et al*. (1967). In sequence, other authors also criticized the stasipatric model of speciation in *Vandiemenella* (Hewitt, 1979; Futuyma and Mayer, 1980). They have centered their criticisms on the difficulty of chromosomal mutants reaching fixation and the plausibility of simpler, allopatric models, once White *et al*. (1967) invoked meiotic drive. In addition, Futuyma and Mayer (1980) argued that stasipatric speciation is unlikely under population genetic theory because it requires the evolution of a new species within the range of an existing one without geographic isolation, once the gene flow between populations would normally prevent the accumulation of genetic differences that could lead to speciation. Furthermore, the observed distribution of chromosome forms does not necessarily imply fixation without the influence of a geographic barrier (Futuyma and Mayer, 1980). 

The second critique was the lack of evidence that the chromosomal rearrangements principally distinguish the chromosomal races that arose within the area of distribution of the parental race in any effective sense. However, the chromosomal, biochemical, mitochondrial, and nuclear molecular data from the *Vandiemenella* populations have shown chromosomal variants in geographic regions, followed by secondary contact, resulting in the presence of a parapatric distribution of chromosomal races (Kawakami et al., 2007a, b, 2009a, b, 2011). Although many authors still consider this dataset more consistent to corroborate allopatric speciation after population fragmentation (Kearney and Hewitt, 2009), there is no way to rule out the reproductive isolation in sympatry generated by chromosomal rearrangements as a primary source of the speciation process.

Recently, genomic analysis bring new clues to this discussion, since the role of transposable elements on chromosomal rearrangements is well known (Klein and O’Neill, 2018), and the species/chromosomal races/cytotypes of *Vandiemenella viatica* analyzed so far indicate tendencies of accumulation of a specific type of repetitive DNA, satellite DNA (Palacios-Gimenez et al., 2020a). Moreover, satellite DNA has been shown to be a good marker of chromosomal evolution among grasshoppers, as seen in the *Schistocerca* genus (Palacios-Gimenez *et al*., 2020b), and could represent an important tool to understand the chromosomal evolution of *V. viatica*. In fact, in virtually all groups of organisms, repetitive DNA plays some role in genomic changes and therefore, karyotypic evolution (Schrader and Schmitz, 2019). 

Several models have been proposed to explain the relationship between chromosome evolution and speciation, particularly regarding the mechanisms of fixation of polymorphisms (for a review see Rieseberg, 2001; Navarro and Barton 2003; Hoffmann and Rieseberg, 2008; Faria and Navarro, 2010; Jackson et al., 2016). However, these models still need to address a practical problem arising from chromosomal speciation: the formation of groups of cryptic species, usually with an intense karyotypic variation. Genomic polymorphisms from hybridisms and events of haploid genome changes may lead to the reorganization of karyotypes, culminating in speciation. In the absence of these events (hybridization and euploidy), the existence of sizable genomic diversity and karyotype plasticity in the ancestral lineage, for example, could explain the emergence of different karyomorphs. These karyomorphs, in turn, can be fixed by evolutionary processes or demographic events, contributing to the formation of new evolutionary significant units (ESUs).

Groups with independent evolutionary units with wide geographic distribution usually constitute informative examples of radiation, since adaptive radiation refers to those evolutionary groups that exhibit an exceptional extension of adaptive diversification in a variety of ecological niches (Schluter, 2000), and non-adaptive radiation can be described as an evolutionary diversification from a common ancestor not accompanied by relevant niche differentiation but by isolation for competition (Gittenberger, 1991). Basically, only mutation and selection processes were sufficient to promote the rapid proliferation of new forms, which supports the theory that compensations in the competitive capacity drive adaptive radiation (Gavrilets and Vose, 2005). On the contrary, niche conservatism can contribute to the rapid accumulation of lineages by promoting the isolation of derived forms and the multiplication of species through a spatially and temporally floating environment (Kozak et al., 2005).

Here, we propose a concept based on the idea of radiation of the karyomorphs to explain the existence of cryptic species karyotypically differentiated, with or without the presence of clines, common in several megadiverse groups of animals and plants.

## The concept of Chromosomal Radiation

We herein introduce the concept of “Chromosomal Radiation,” which explains the diversity patterns observed for many years in the chromosomal studies of various diploid organisms. This concept can be applied to studying any group of cryptic species, chronospecies, or sister species, which display lower or higher molecular and usually low morphological divergence, associated with large karyotype diversity, i.e., a rapid chromosome evolution. 

**Figure 1 -:**
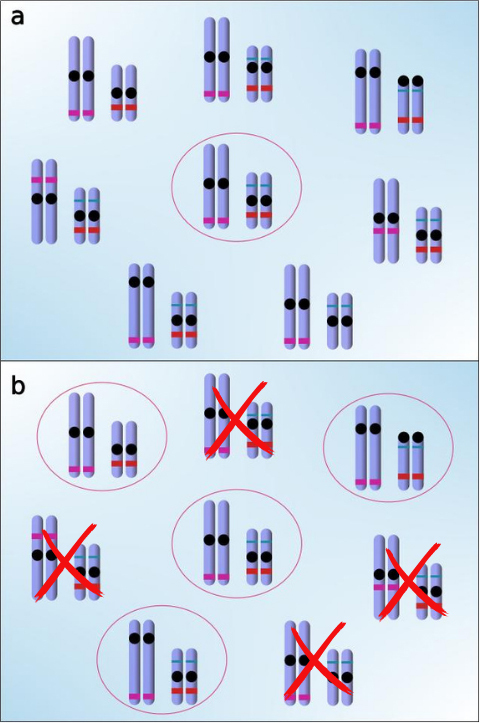
The schema delineates the process of chromosomal radiation through two sequential stages. Firstly, Stage a) involves the emergence of populations presenting rearrangements from an ancestor karyotype exhibiting karyotypic plasticity, resulting in intrapopulation genetic variability. These rearrangements include both major structural alterations and minor polymorphisms such as heterochromatin or ribosomal sites (not shown in the schema). Subsequently, Stage b) encompasses the independent spread and fixation of various rearrangements in distinct populations over time, concomitant with the extinction of other rearrangements. This stage culminates in interpopulation variability. Since not all possible polymorphisms are generated or fixed, and other subsequent rearrangements may arise and reorganize the genome of mutant individuals, several variant forms are observed in natural populations.

We believe, therefore, that chromosomal radiation consists of a pattern in which the common ancestor of the group has the potential for karyotype plasticity. Just as phenotypic plasticity refers to the ability of the same genotype to express different phenotypic characteristics in different environments (West-Eberhard, 1989; Whitman and Agrawal, 2009; Secer et al., 2022), karyotype or chromosomal plasticity represents the capacity of the same (or near same) phenotype to exhibit different chromosomal characteristics in different environments and/or populations. In *Leishmania*, for instance, the tolerance of natural populations to different types of aneuploidies appears to be crucial for the homozygosity (monozygosity) of distinct genes, thereby constituting a significant tool for investigations concerning gene function and regulatory mechanisms (Cruz et al., 1993; Dujardin et al., 1995). With over 70 chromosomal races (Wójcik et al., 2003), *Sorex araneus* stands as a significant example of how a species can modify its chromosomes without substantial phenotypic manifestations, although certain correlations between morphometrics and karyotypes can be observed in specific groups (Polly, 2007). Furthermore, processes of chromosome gain and loss associated with equid evolution underscore the importance of karyotypic plasticity in evolution (Jónsson et al., 2014). Such karyotype plasticity enables the expansion of forms, with new populations presenting different karyotypes, which can be set independently by evolutionary and/or demographic processes. Under this explanation, chromosomal differentiation currently found in populations that do not always show signs of homology would be the result of two main processes: (i) the emergence of rearrangements that generate intrapopulation genetic variability in different lineages, without affecting the reproductive performance of individuals (Figure 1a); and (ii) the spread and fixation of different rearrangements independently in different populations over time, leading to the interpopulation variability observed (Figure 1b).

Thus, cryptic species complexes where there are a large diversity of karyomorphs with autapomorphies must have originated due to plasticity in the ancestral karyomorph. The multiple karyotypic forms found in cryptic species could constitute, therefore, important examples of non-adaptive or adaptive chromosomal radiation, depending on the relation of the character with the environment.

Despite the debates about the role of chromosomal rearrangements in the diversification of species, there is evidence that unbalanced rearrangements can interfere in gametogenesis, reinforcing the reproductive isolation of karyomorphs by reduction of gene flow, as some species have increased tolerance to chromosomal rearrangements, maintaining polymorphic populations or possessing large karyotype plasticity (Pazza et al., 2018). Regardless of the explanations for the appearance and fixation of the existing variation, there is a consensus that chromosomes play an important role in the speciation of several groups of organisms. 

In theory, although frequently a causal factor of differentiation, the accumulation of chromosomal rearrangements can also be the result of evolutionary processes in natural populations. In groups where gene flow persists for a long time between different forms of a lineage, it is very difficult to assign reproductive isolation to chromosomal rearrangements. In species with large karyotype plasticity, for example, equids (Jónsson et al., 2014), the maintenance of gene flow between different karyomorphs appears to be possible, at least for some time. This may occur more often than we estimate, explaining the discontinuity between molecular and karyotypic evolution seen in some groups with significant chromosomal diversity (Kavalco et al., 2016). 

The pattern observed among populations originating from chromosomal radiation would be similar to that seen in a wide range of species. Almost all living organisms possess some level of karyotype plasticity, although, in certain groups, large plasticity can be considered a characteristic biological trait. This is the case in several groups of fish, for example.

## Chromosomal radiation in fish

Chromosomal data had long supported the idea that the genus *Astyanax* (Teleostei, Characiformes) was polyphyletic, as noted by Weitzman and Malabarba (1998), and had even revealed the existence of several cryptic species within the group, as summarized by Pasa and Kavalco (2007). More recently, molecular studies have provided further evidence for the formation of structured clades within *Astyanax* (Mello et al., 2015; Rossini et al., 2016; Pazza et al., 2018; Pasa *et al*., 2021), and it has become clear that chromosomal features are linked to these clades, especially with regard to the origin and spread of the As51 satellite DNA (Mestriner et al., 2000) and the localization of the 5S ribosomal gene. Additionally, Pazza *et al*. (2018) highlighted the association between these chromosomal features and the species’ phylogeny. This was reflected in a revision by Terán et al. (2020), which involved the reassignment of some *Astyanax* species to six different genera, reverberating the molecular phylogenies and the chromosomal features described. For instance, *Astyanax* species from coastal river basins of Brazil were now assigned to the genus *Deuterodon* (such as *D. hastatus*, *D. giton* and *D. intermedius*), which lacks the As51 satellite DNA and in which the rDNA 5S is located only on distal position on ST/A chromosomes (Kavalco *et al*., 2004, 2009; Rodrigues-Oliveira et al., 2023). Meanwhile, traditional species complexes such as *Astyanax fasciatus* and *Astyanax scabripinnis* were moved to the genus *Psalidodon*, which has high levels of karyotypic diversity and chromosomal rearrangements, where the As51 satellite DNA plays an important role (Kavalco *et al*., 2013). So, we have the emergence of a motor for karyotype plasticity, and the spread of a high diversity of karyotypes of low genetic distance in a short time (Pazza *et al*., 2018), i.e., chromosomal radiation. 

The phenomenon of dispersion of repetitive sequences within chromosomes is often attributed to the activity of transposable elements (TE) present in the genomes (Silva-Neto et al., 2015). Such activity may explain the heterogeneous patterns observed in the distribution of heterochromatin blocks and rDNAs 5S cistrons across different pairs of chromosomes. Evidence from rDNA sequence analyses indicates the presence of these elements within spacer regions, facilitating the dissemination of gene families across functional copies or pseudogenes (Drouin and de Sá, 1995; Gornung, 2013; Rebordinos et al., 2013; Symonová et al., 2013). The dispersion of TEs (and consequently of ribosomal DNA, for example) could then affect the rate of recombination in the genomes and lead to rapid divergence of the karyotype/genome, as observed in the salmonids *Coregonus albula* and *Coregonus fontanae* (Symonová *et al*., 2013). TEs have faced strong selection against them due to unequal homologous recombination, which can lead to their elimination from a genome or the production of inviable chromosomal aberrations (Schrader and Schmitz, 2019). Despite this, TEs have accumulated in most eukaryotic genomes. This raises the question of whether the evolution of epigenetic silencing mechanisms controlling recombination played a crucial role in enabling the invasion of TEs into eukaryotic genomes, as suggested by Fedoroff (2012).

In turn, the species *H. ancistroides* (Siluriformes) has an extensive diversity of karyomorphs with markers showing homoplastic phenotypes, without clina across geographic distribution, and between haplogroups. One explanation for this pattern is that the period of diversification and the time spent during population divergence was so brief that some karyomorphs and haplotypes may have been lost (Rocha-Reis et al., 2021). Such losses difficult the understanding the entire evolutionary landscape of the group, mainly due to the lack of intermediate forms. However, the 19 karyomorphs of *H. ancistroides* are so exclusive that each population has a particular cytotype (Rocha-Reis *et al*., 2018) and two populations have shown different Sexual Chromosome Systems, with male (Rocha-Reis *et al*., 2018) and female (Lara Kamei et al., 2017) heterogamety. Comparing the data from the morphology, chromosome macrostructure, genetic and genomic markers, plus the mitochondrial differences, Rocha-Reis *et al*. (2020) argue that *H.* aff. *ancistroides* with XX/XY and 2n=66 need to be formally described and named as a taxonomically valid species.

This was also observed in species of *Chromaphyosemion* (Cyprinodontiformes), especially in *C. riggenbachi* (Völker et al., 2006). The authors emphasize that karyotypic and haplotypic differentiation suggests speciation in its initial stages and that the karyotype differentiation in *C. riggenbachi* is an ongoing process, in which the rearrangements may be fixed by several processes such as natural selection, genetic drift, or meiotic impulse. According to Völker *et al*. (2008), various factors, such as the accumulation of chromosomal and genetic incompatibilities, as well as sexual selection, are responsible for driving speciation in *Chromaphyosemion*. It is not necessary that the relative potency of these factors remains constant in all speciation processes within the group, and in some instances, interactions among them, such as reinforcement, may take place (Völker *et al*., 2008).

## Chromosomal radiation in other vertebrates

In other vertebrates, such as amphibians, reptiles, and mammals, large diversity associated with specious groups with wide distribution can also be observed. Rodents are known to constitute a group with a sizable chromosomal diversity and several polymorphisms. At least seven cryptic species are observed in the African gerbil *Taterillus* spp. (Dobigny et al., 2001), distinguishable only by their chromosomal characteristics, with karyotypes displaying extensive chromosomal rearrangements (Dobigny *et al*., 2002a, b). Dobigny *et al*. (2005) have linked the observed rearrangements to the association between allopatry and bottlenecks due to drastic climate change, and they assume that the *Taterillus* genome is (or has recently been) particularly plastic and may consequently have a high probability of chromosomal mutation. Aniskin et al. (2005), in turn, quote as a remarkable feature of some gerbils’ genomes, is the accumulation of a high heterogeneous constitutive heterochromatin on lineages. In fact, the chromosomal changes and the amount of heterochromatin observed in species of the subgenus *Gerbillus* were highlighted through the utilization of a molecular phylogeny based on *cyt b*, where different cytotypes should be regarded as traits that have evolved over time, connecting the accumulation of heterochromatin with the presence of rearrangements (Abiadh et al., 2010).

In Neotropical Rodentia, extensive Robertsonian rearrangements, tandem fusions, fissions, and peri and paracentric inversions, besides heterochromatin polymorphisms, have been described. A review compiling data on the role of rearrangements in speciation and cytotaxonomy of South American species and demonstrating the richness of distinct chromosomal forms makes it possible to delimit cryptic species in *Akodon*, *Calomys*, *Cerradomys*, *Euryoryzomys*, *Delomys*, *Hylaeamys*, *Juliomys*, *Neacomys*, *Oecomys*, *Oligoryzomys*, *Ctenomys*, *Thrichomys*, and *Trinomys* was presented by Di-Nizo et al. (2017). 

Oliveira da Silva et al. (2019) suggest that the process of chromosome evolution in *Neacomys* may be more intricate and involve more events than initially anticipated, based on the findings of phylogenetic relationships and chromosomal signatures. Their paper presents not only a populational phylogenetic tree with the variant karyotypes but a chromosomal painting showing the homologies among the chromosomes and rearrangements. Do Nascimento Moreira *et al*. (2022) suggest that genomic components, such as repetitive DNA, stimulate karyotype diversity in rodents belonging to the Oryzomyini tribe and contribute to their chromosomal variability. 

In the case of *Ctenomys*, the most diverse mammals at the species level, the correlation between species diversity and chromosomal variability is not straightforward, as various chromosome sets have been documented within the same species, whereas different species may share similar forms of complex rearrangements (Buschiazzo et al., 2022). According to the authors, the diversity in chromosomes observed in *Ctenomys* and the variation in the prevalence of specific types of chromosome rearrangements among different groups of species suggest distinct patterns of diversification within each lineage. Therefore, to justify the significant differences in the structure of chromosomes, even within the same species, it is necessary to consider an increased rate of chromosome evolution (Buschiazzo *et al*., 2022).

It is undeniable that the ancestors of these rodents had the potential for karyotypic plasticity since so many different “types” arose from the preexisting chromosome variation of natural populations. Thus, we see clear radiation of karyomorphs, most probably due to the pre-zygotic isolation that the chromosomal alterations may have caused.

## Chromosomal radiation in *Drosophila*

Another group with significant chromosomal diversity and where radiation probably played an important evolutionary role is the genus of arthropods *Drosophila*. Dobzhansky had already described polymorphisms in Drosophila chromosomes in the 1930s (Dobzhansky, 1947), since the first description of chromosome rearrangements, causing lack or recombination, by Sturtevant (1917). In the 1960s more than 80 different karyomorphs were recognized as acting in the diversification of Hawaiian *Drosophila* spp., with phylogenies based on chromosomal rearrangements of the group since then (White, 1973). It seems a lot, but this number may be several-fold higher since the adaptive radiation that led to the speciation in these islands has generated over 700 species and the main chromosomal markers emerged after the 1960s. 

There are two mechanisms explaining the origin of inversions in Drosophila. The first and predominant mechanism is ectopic recombination between repetitive sequences in genomic regions prone to breakage, resulting in the inversion of the sequence. The second mechanism is based on two staggered double-strand breaks around the future inversion breakpoints, which leads to the reinsertion of the inverted segment and the generation of duplications around the breakpoints (Kapun and Flatt, 2019). It is worth noting that Drosophila species exhibit varying degrees of tolerance towards inversion polymorphisms. For instance, sister species of *D. melanogaster*, such as *D. simulans*, *D. mauritiana*, and *D. sechellia*, are virtually devoid of inversions, with only a few cases of unique inversion polymorphisms recorded at low frequencies in natural populations (see Kapun and Flatt, 2019 for a review). This lack of inversions in these species may be attributed to a lower number of transposable elements (TEs), which are known to play a crucial role in the generation of inversions, and/or larger population sizes as compared to *D. melanogaster*, leading to distinct patterns of genetic variation (Aquadro et al., 1988). Similarly, the chromosomal variations described in groups of sister species or cryptic species of many other insects, including beetles, locusts, crickets, and mosquitoes are historically extensive. 

## Chromosomal radiation in plants

Chromosomal diversity is not exclusive to animals. In plants, besides hybridisms, numerical and structural polymorphisms, and polyploidy events are implicated in the rapid speciation of various groups. In Liliaceae, there is evidence of fusions, fissions, translocations, and inversions that generate a great diversity of karyomorphs in several genera, altering karyotypic symmetry (Peruzzi et al., 2009). Karyomorph radiation from an ancestor of high intrinsic diversity, naturally selected or fixed by genetic drift, explains all these patterns. 

Similarly, chromosomal diversity in the Asteraceae is substantial and seen in several genera, especially in the patterns of heterochromatin, where differences in the sizes and number of C+ bands appear to be related to the presence of rearrangements (see Marinho et al., 2017). Repetitive DNA is generally the most variable and rapidly changing part of the genome, with significant differences in both the sequence and the number of individual motifs between species (Heslop-Harrison and Schwarzacher, 2007). The idea that several derived karyomorphs can be produced from one pluripotent karyomorph as a result of the intrinsic karyotype plasticity of the species is more parsimonious, there being multiple possible forms for each character, and in the case of chromosomes carrying the sites of heterochromatin they are not necessarily homologous to the ancestral karyomorph. That is, each new arrangement could raise independently, creating several new lineages from the same direct ancestor. This would explain not only the distribution pattern of this type of DNA but also the existence of different karyotype formulas and heterochromatic blocks not shared between populations.

In Orchidaceae, one of the most numerous angiosperm families, groups of sister species and cryptic species feature a large number of karyomorphs, such as *Epidendrum* (Nóbrega et al., 2017). The presence of repetitive elements such as transposable elements, satellites, and others, can result in variations in the distribution of heterochromatin and in the genome size, reflected in the phylogenetic lineages (Pessoa et al., 2021). The absence of blocks in one of the homologs reveals the likely occurrence of unequal exchanges during cell division, where a part or the entire heterochromatin block is translocated to another chromosome. Interestingly, such polymorphisms are observed, especially in hetero-karyotypes, since the major part of the polymorphisms appears in heterozygosity in populations, and, depending on demographic events and evolutionary processes, can be fixed or eliminated over generations, usually after overcoming subdominance (Hoffmann and Rieseberg, 2008; Kirkpatrick, 2010) or by selection or genetic drift in small populations (Spirito, 1998). This indicates not only that there is variation in the population, but it is in the overt process of chromosome evolution. 

Polyploidy is considered a paramount adaptive mechanism in chromosomal evolution and speciation. The analysis of meiotic chromosome pairing is a valuable tool in studying polyploids, but it is subject to the influence of genes that guarantee the exclusive pairing of homologous chromosomes (Heslop-Harrison et al., 2023). In order to prevent the occurrence of multivalents during meiosis, which can lead to non-disjunction of chromosomes and the production of infertile gametes, polyploids have evolved genetic mechanisms, such as the Ph-locus in wheat, that promote proper chromosome pairing during meiosis (Rawale et al., 2019). These mechanisms help to restore diploid behavior in the polyploid (Heslop-Harrison *et al*., 2023). Other means of dissemination, such as apomictic seed production or vegetative propagation, permit the propagation of species without the need for conventional meiosis. These mechanisms also enable the survival of taxa with an odd number of ancestral genomes, such as the triploid dessert banana *Musa acuminata* (Heslop-Harrison and Schwarzacher, 2007), and can help the polymorphism to spread to surrounding populations and achieve fixation.

## Adaptive and non-adaptive chromosomal radiation

Several diversifications may contain elements of both adaptive and non-adaptive radiation (Rundell and Price, 2009). Thus, it seems quite plausible that we can identify adaptive and non-adaptive traits in karyotypes of groups of sister species and accept that chromosomal radiation can cover both adaptive and non-adaptive characters, not being mutually exclusive. Chromosomal inversions have been shown to play a significant role in a local adaptation by capturing multiple linked variants that confer adaptive benefits within the specific environment. The heterozygosity of inverted regions suppresses recombination due to the production of unbalanced gametes or the inability of inverted regions to synapse (Hoffmann and Rieseberg, 2008; Kirkpatrick, 2010). Consequently, inversions help maintain linkage disequilibrium among a group of locally adapted variants in the presence of gene flow and migration from populations without the inversion (Kirkpatrick and Barton, 2006; Hoffmann and Rieseberg, 2008; Kirkpatrick, 2010). The selective advantage of inversion is not dependent on epistatic interactions between adaptive variants, and their effects can be additive (Kirkpatrick and Barton, 2006). Furthermore, it is possible that the breakpoints of inversions may have phenotypic effects and, therefore, be adaptive. Additional adaptive mutations may or may not occur within the inversion (Kirkpatrick and Barton, 2006).

The disproportionate influence of sex chromosomes on the speciation process has been widely acknowledged. The fixation rate of X-linked inversions and their polymorphism levels are often observed to be higher compared to autosomal inversions in several insect species. Cheng and Kirkpatrick (2019) report that X-linked inversions in *Drosophila* capture a significantly larger number of genes (67% more) compared to autosomal inversions. Similarly, the genetic differentiation between pairs of populations or species in birds is typically greater on the sex chromosomes when compared to the autosomes (Hooper et al., 2019). 

## Conclusion

The findings of this study provide substantial support for the notion that certain groups of organisms exhibit intrinsic genome-based karyotype plasticity. The observed widespread occurrence of cryptic evolutionary units correlates with a pattern akin to species radiation, termed here as “chromosomal radiation.” This phenomenon can manifest in both adaptive scenarios, wherein distinct karyotypes enable coping with selective environmental pressures, as well as non-adaptive scenarios, where karyomorphs are maintained in small populations through the process like genetic drift.

The recognition of the concept developed in our article may facilitate the discussion of topics related not only to evolution and karyotypic speciation among or within populations but also to chromosomal evolution itself, including the processes of emergence of variant karyomorphs and specialized chromosomes, such as sex chromosomes, in an eco-evolutionary context.
